# 2281: Temperature regulating wheelchair cushion for prevention of pressure ulcers

**DOI:** 10.1017/cts.2017.217

**Published:** 2018-05-10

**Authors:** Metin Yavuz, Ali Ersen, Linda Adams

## Abstract

OBJECTIVES/SPECIFIC AIMS: According to the US census, there are 3.3 million Americans who have to use wheelchairs in order to maintain their mobility. About 50% of these patients develop a pressure ulcer at some point during their life time. Three major factors contribute to pressure ulceration; pressure, tissue temperature, and maceration due to sweating. The objective of this study is to develop a temperature regulating wheelchair cushion in order to address elevated tissue temperatures and related sweating. METHODS/STUDY POPULATION: We instrumented a wheelchair with cooling elements, a water filled cushion and a pump. The pump moves the water through the cooling elements where water temperature drops down to 15°C. The water then moves to the cushion where it cools the tissue and then back to the cooling elements. RESULTS/ANTICIPATED RESULTS: We recruited 1 healthy subject to sit on the instrumented wheelchair and then obtained thermographs of the cushion surface using an infrared thermal camera. After 1 minute of sitting on the cushion the minimum temperature was recorded as 27°C. After 10 minutes the temperature dropped to 23.3°C. DISCUSSION/SIGNIFICANCE OF IMPACT: In this ongoing proof-of-concept study we are investigating if circulating chilled water inside a wheelchair cushion is a feasible method to regulate tissue temperatures at the 25–28°C range. This range has been shown to delay ulceration under loading conditions that simulate sitting on a wheelchair. Initial results indicate that this may be an effective ulcer prevention method.

